# Indoor air pollution exposure from use of indoor stoves and fireplaces in association with breast cancer: a case-control study

**DOI:** 10.1186/1476-069X-13-108

**Published:** 2014-12-12

**Authors:** Alexandra J White, Susan L Teitelbaum, Steven D Stellman, Jan Beyea, Susan E Steck, Irina Mordukhovich, Kathleen M McCarty, Jiyoung Ahn, Pavel Rossner, Regina M Santella, Marilie D Gammon

**Affiliations:** Department of Epidemiology, University of North Carolina, CB#7435, McGavran-Greenberg Hall, Chapel Hill, NC 27599-7435 USA; Department of Preventative Medicine, Mt. Sinai School of Medicine, New York, NY USA; Department of Epidemiology, Mailman School of Public Health, Columbia University, New York, NY USA; Consulting in the Public Interest (CIPI), Lambertville, NJ USA; Department of Epidemiology and Biostatistics, University of South Carolina, Columbia, SC USA; Exposure, Epidemiology and Risk Program, Department of Environmental Health, Harvard School of Public Health, Boston, MA USA; Department of Population Health, New York University School of Medicine, New York, NY USA; Department of Genetic Ecotoxicology, Institute of Experimental Medicine ASCR, Prague, Czech Republic; Department of Environmental Health Sciences, Columbia University, New York, NY USA

**Keywords:** Polycyclic aromatic hydrocarbons, GST, p53, Cancer, Breast

## Abstract

**Background:**

Previous studies suggest that polycyclic aromatic hydrocarbons (PAHs) may adversely affect breast cancer risk. Indoor air pollution from use of indoor stoves and/or fireplaces is an important source of ambient PAH exposure. However, the association between indoor stove/fireplace use and breast cancer risk is unknown. We hypothesized that indoor stove/fireplace use in a Long Island, New York study population would be positively associated with breast cancer and differ by material burned, and the duration and timing of exposure. We also hypothesized that the association would vary by breast cancer subtype defined by p53 mutation status, and interact with glutathione S-transferases GSTM1, T1, A1 and P1 polymorphisms.

**Methods:**

Population-based, case-control resources (1,508 cases/1,556 controls) were used to conduct unconditional logistic regression to estimate adjusted odds ratios (OR) and 95% confidence intervals (CI).

**Results:**

Breast cancer risk was increased among women reporting ever burning synthetic logs (which may also contain wood) in their homes (OR = 1.42, 95% CI 1.11, 1.84), but not for ever burning wood alone (OR = 0.93, 95% CI 0.77, 1.12). For synthetic log use, longer duration >7 years, older age at exposure (>20 years; OR = 1.65, 95% CI 1.02, 2.67) and 2 or more variants in GSTM1, T1, A1 or P1 (OR = 1.71, 95% CI 1.09, 2.69) were associated with increased risk.

**Conclusions:**

Burning wood or synthetic logs are both indoor PAH exposure sources; however, positive associations were only observed for burning synthetic logs, which was stronger for longer exposures, adult exposures, and those with multiple GST variant genotypes. Therefore, our results should be interpreted with care and require replication.

**Electronic supplementary material:**

The online version of this article (doi:10.1186/1476-069X-13-108) contains supplementary material, which is available to authorized users.

## Background

Breast cancer is the most common cancer diagnosed among women in the United States (U.S.) [1]. Experimental research indicates that polycyclic aromatic hydrocarbons (PAHs) increase development of mammary tumors [2]. The association between PAHs and human breast cancer, however, remains unclear [3].

PAHs are formed from the incomplete combustion of organic material [4]. The main sources of ambient PAH exposure in the general population include tobacco smoke, outdoor air pollution, indoor stoves and/or fireplaces, and the intake of grilled and smoked foods[2]. PAHs are established carcinogens to the lung and may also be carcinogenic to the mammary gland [5].

Previous studies have often relied on measuring PAH exposure with the biomarker PAH-DNA adducts [6–9]. However, this body burden measure likely reflects a host’s susceptibility to PAH as well as exposure levels [3]. Also, adducts represent PAH exposure in the short-term (e.g., several months to several years), which may not be the relevant time period as breast cancer is thought to develop over many years [10]. There is no PAH biomarker that reflects longer-term (e.g., more than a few years) exposure to PAH sources [10].

Indoor air pollution, or household air pollution, from indoor stoves/fireplaces is an important ambient PAH source and is of significant world-wide public health concern [5]. Previous research on indoor air pollution from solid fuel burning has predominantly focused on respiratory health effects or cancer outcomes in developing countries where exposure levels are high compared to the U.S. [11]. Indoor air pollution from burning of wood or coal has been associated with cancers of the lung in an international pooled study [12] and upper aero-digestive tract in India [13], but no studies thus far have investigated the association between indoor air pollution or stove/fireplace use with breast cancer [11]. Open fireplaces in the home have been associated with bulky DNA adduct levels [14] which are known to be relevant for breast cancer risk [6–9].

Environmental exposures that occur early in life, or during hypothesized biological windows of susceptibility may be more strongly associated with the risk of breast cancer [15]. For example, previous breast cancer research has suggested that exposure to ionizing radiation is most important before age 20 years [16]. However, it is unknown whether PAH exposure during breast development is associated with subsequent breast cancer risk [15].

Specific base substitutions and other transitions and transversions in the tumor suppressor gene p53 have been associated with PAH exposure [17]. Thus, it is plausible that the association with PAH exposure may be evident when we consider breast cancer subtype defined by p53 mutation status of the index tumor. Similarly, because PAH have been shown to have estrogenic properties [18], the association with indoor stove/fireplace use may be more pronounced among certain breast cancer subtypes defined by hormone receptor status.

Glutathione S-transferase (GST) enzymes aid in the metabolism of PAHs and polymorphisms in the GST genes may alter individual’s ability to metabolize PAH compounds, and to remove reactive intermediates from the body [19]. GST variants are hypothesized to interact with PAH exposures, although previous research has been inconclusive on a possible interaction between GSTs and PAH with respect to breast cancer [20, 21].

Here we report on the association between use of indoor stoves and fireplaces and breast cancer risk. We hypothesized that this association would be modestly elevated for all types of material burned, stronger with increasing years of exposure and for early-life exposure and that it may vary by the timing of exposure and among susceptible subgroups defined by GST gene variants, hormone receptor subtype and p53 tumor mutations.

## Methods

Our investigation builds upon population-based case-control resources from the Long Island Breast Cancer Study Project (LIBCSP). Parent study methods have been previously described in detail [22]. IRB approval was obtained from all participating institutions.

### Study population

The LIBCSP included English-speaking women residing in Nassau and Suffolk counties on Long Island, New York. Cases were women newly diagnosed with a first primary in situ or invasive breast cancer between August 1, 1996 and July 31, 1997. Cases were ascertained by daily/weekly contact with pathology departments of all 28 hospitals on Long Island and three tertiary care hospitals in New York City.

Controls were randomly selected female residents from the two source counties who had no history of breast cancer and were frequency matched to cases based on the expected case age distribution by 5-year age group. Potential controls were identified by random digit dialing for those less than 65 years of age and by Health Care Finance Administration rosters for those 65 years and older. The response rate for women <65 years was 76.1% and for women >65 years was 43.3% [22].

Written signed informed consent was obtained from all participants prior to interview. A total of 1,508 cases and 1,556 controls (82% and 62.7%, respectively, of all eligible subjects) completed the interview. Participants ranged in age from 20 to 98 years, with one third 65 years of age or older at the time of diagnosis (for cases) or identification (for controls), and 67% were postmenopausal. In addition, 94% self-identified as white, 5% black, and 2% other, which reflects the underlying racial distribution of these two Long Island counties at the time the study was conducted.

### Questionnaire assessment of indoor air pollution from stoves/fireplaces

Indoor stove/fireplace use was assessed using a structured questionnaire. The LIBCSP case-control interview occurred in the respondents’ homes and, for cases, within several months of the diagnosis of the first primary breast cancer. All participants were administered the main case-control questionnaire, a comprehensive assessment of known and suspected risk factors for breast cancer. This instrument included a question asking whether a participant used an indoor stove or fireplace ≥3 times a year for each residence lived at on Long Island; and, if yes, whether they burned wood, coal, gas and/or synthetic logs. Synthetic logs also contain wood; therefore, it is important to note that future references to wood-burning exclude synthetic logs use. The date and/or ages of participants at time of living in each residence was used to calculate exposure duration and timing. Exposure frequency or exposures to stove/fireplace use at non-residences or residences outside of Long Island were not captured.

### Tumor p53 mutation analysis

To consider breast cancer subtype, we utilized data on p53 mutation status of the tumor. Archived paraffin-embedded tumor tissue was obtained and DNA was extracted (n = 859). Mutations were detected in exons 5-8 of p53, described in detail in Rossner et al. [23]. Samples were amplified using polymerase chain reaction (PCR) and the Surveyor Mutation Detection Kit (Transgenomic, Omaha, NE, USA) was used to screen for p53 mutations. Positively identified samples were confirmed and identified using PCR amplification and sequencing using an ABI 3100 capillary sequencer (Applied Biosystems Inc., Foster City, CA, USA).

### Hormone receptor subtype

Breast cancer subtype defined by hormone receptor status was obtained from medical records [22]. Cases (97.7%) signed a medical record release form for the abstraction of clinical characteristics of breast cancer. For 95.2% of cases, medical records were successfully located and abstracted. Hormone receptor status of the first primary breast cancer was available from the medical record for 990 cases (65.6%).

### GST laboratory assays

To consider potential interactions between indoor stove/fireplace exposure and GST polymorphisms, we utilized genotyping data, which was assayed as follows. Blood samples were obtained from 73% of cases and controls [22] and DNA was isolated. GSTM1 and GSTT1 were genotyped using multiplex polymerase chain reaction method [24]. GSTP1 Ile105Val (rs1695) and GSTA1 (three linked based substitutions in promoter at -567, -69 and -52) genotyping were completed using high-throughput MALDI-TOF [24, 25].

### Other covariate assessment

To consider potential confounders and/or effect modifiers, responses to other sections of the questionnaire, including reproductive and menstrual histories, education, life course body size, cigarette smoking and race, were used. Distributions of known risk factors among the LIBCSP population have been described [22].

### Statistical analyses

We undertook four analytic steps: (1) estimated the association between breast cancer risk and residential indoor stove/fireplace use, with consideration given to potential confounders; (2) explored associations with more detailed exposure information, including material burned and timing of exposure; (3) explored associations with breast cancer subtype; and (4) considered effect modification by GST polymorphisms, menopausal status and smoking history.

First, we used unconditional logistic regression [26] to estimate odds ratios (ORs) and 95% confidence intervals (95%CIs) for associations between breast cancer and ever using an indoor stove/fireplace at a Long Island residence. All statistical models were adjusted for the frequency matching factor, 5-year age group. Other potential confounders were identified from a directed acyclic graph [27] and included parity, education, marital status, active/passive smoking, age at menarche, religion, race, lifetime alcohol intake, physical activity (total average hours/week), body mass index, history of lactation, age at first birth, exogenous hormone use, and family history of breast cancer. Both multi-variable adjusted and age-adjusted models are included in the results. All analyses were completed using SAS 9.2 (Cary, NC).

Second, associations between breast cancer risk and detailed exposure characteristics, including stove/fireplace type, duration, and timing, were also considered using unconditional logistic regression. For example, we used indicator variables to assess the association for each material burned (wood, synthetic logs, coal or gas); subanalyses, however, were limited to wood and synthetic logs due to the limited sample sizes of women reporting burning coal or gas. To assess possible correlation between women who reported burning wood and those who reported burning synthetic logs, a kappa statistic was calculated [28]. We used information on the time spent living in a Long Island residence where an indoor stove/fireplace was ever used to determine an estimate of exposure duration; categories were a priori defined based on quartiles among the controls and women with no stove/fireplace use were the referent group. Separate models were fitted for any stove/fireplace use, wood burning and synthetic log burning. Tests for trend were calculated using a Mantel test statistic for the continuous variable [29]. When examining associations for exposures <20 vs ≥20 years of age, analyses were restricted to women who lived on Long Island prior to age 20 years, given LIBCSP participants were only queried about their residences on Long Island. We considered whether an indoor stove/fireplace was used during a possibly biologically susceptible period, <20 years of age (exposed participants could be classified as exposed for <20 years of age, ≥20 years of age or both); timing was further assessed by whether the participant specifically burned wood or synthetic logs.

Third, associations with the cases categorized by breast cancer subtypes, defined by p53 mutation status and hormone receptor status, were examined using polytomous logistic regression [30], which simultaneously yields effect estimates for the associations among multiple case groups (e.g., multiple breast cancer subtypes) and a single control group. The ratio of the ORs and 95% CI for associations were used as a measure of heterogeneity [31] when comparing the ORs among breast cancer subtypes, defined either by p53 mutation status (p53+ vs. p53-), or combined estrogen receptor progesterone receptor (ERPR) status (ER+ PR+ vs. all other subtypes, and ER-PR- vs. all other subtypes). We performed additional analyses using a case-case approach [32] with age-adjusted unconditional logistic regression to investigate associations between p53 mutation type and indoor stove/fireplace use, stratifying by burning either wood or synthetic logs.

Fourth, to consider possible effect modification by GST polymorphisms, menopausal status or active/passive smoking status, unconditional logistic regression was used. Effect measure modification on a multiplicative scale was assessed by comparing multivariable models with and without cross-product terms of the exposure and menopausal status (pre-, post-menopause) and active/passive smoking (neither active nor passive, passive only, active only, active and passive) as well as using the approach described below for each GST polymorphism separately, as well as number of GST variants [30]. For possible modification on the additive scale, interaction contrast ratios (ICRs) and 95% confidence intervals were calculated [33]. GSTM1 and GSTT1 genotypes were classified as ‘present’ or ‘null’ as determined by the presence or absence of the allele, using a dominant model approach. For GSTP1, the AA genotype was considered common and either AG or GG genotypes were considered variants. For GSTA1*A and GSTA1*B genetic polymorphism, GSTA1*B was classified as the variant and GSTA1*A as the common polymorphism [20, 25]. The reference group for each polymorphism was defined as GSTT1 present, GSTM1 present, GSTP1 common and GSTA1 common. We considered each polymorphism separately and also by an individual’s number of variant alleles (<2 variants, ≥2 variants), as previously described [20].

## Results

Case-control participant characteristics, stratified by any stove/fireplace use, are displayed in Table 1. Distributions of stove/fireplace users and non-users were similar across demographic and breast cancer risk factors.

**Table 1:** **Case-control participant characteristics, stratified by indoor stove/fireplace use, among Long Island, NY women, LIBCSP 1996-1997**

	Stove/fireplace non-users				Stove/fireplace users			
Characteristics	Cases		Controls		Cases		Controls	
***Categorical variables***	N	%	N	%	N	%	N	%
Age								
<35	20	2.6	19	2.4	18	2.4	26	3.4
35-44	77	10.2	100	12.8	104	13.9	144	18.8
45-54	149	19.7	185	23.7	248	33.2	237	30.9
55-64	191	25.2	221	28.3	181	24.2	181	23.6
65-74	217	28.7	170	21.8	146	19.5	138	18.0
75+	103	13.6	86	11.0	50	6.7	42	5.5
Education								
< High school	132	17.6	103	13.2	50	6.7	46	6.0
High school graduate	308	41.0	309	39.7	228	30.6	215	28.0
Some college	158	21.0	185	23.8	201	26.9	228	29.7
College graduate	72	9.6	98	12.6	119	16.0	138	18.0
Post-college education	82	10.9	84	10.8	148	19.8	140	18.3
Religion								
Protestant	166	22.0	168	21.6	193	25.8	205	26.7
Catholic	435	57.7	468	60.1	422	56.5	445	57.9
Jewish	134	17.8	128	16.4	124	16.6	108	14.1
None or other	19	2.53	15	1.93	8	1.07	10	1.3
Race								
White	691	91.6	695	89.0	717	96.0	728	94.8
Black	47	6.2	64	8.2	21	2.8	20	2.6
Other	16	2.1	22	2.8	9	1.2	20	2.6
Marital status								
Never	38	5.0	41	5.3	26	3.5	28	3.7
Ever	718	95.0	740	94.8	721	96.5	740	96.4
Lifetime alcohol intake								
Non-drinkers	356	47.0	366	47.0	240	32.1	233	30.4
<15 gram/day	318	42.0	320	41.1	372	49.8	414	54.0
15-30 gram/day	55	7.3	47	6.0	91	12.2	72	9.4
>30 gram/day	28	3.7	46	5.9	44	5.9	48	6.3
Physical activity (hours/week)								
0	258	35.7	256	35.1	163	23.5	156	21.6
<0.69	170	23.6	151	20.7	168	24.2	181	25.0
0.7-2.6	153	21.2	169	23.2	178	25.6	197	27.2
≥2.6	141	19.5	154	21.1	186	26.8	190	26.2
Smoking history								
Neither active nor passive	108	14.7	110	14.5	58	7.9	72	9.6
Ever passive	228	31.1	270	35.7	254	34.6	224	29.9
Ever active	77	10.5	66	8.7	58	7.9	77	10.3
Both active and passive	320	43.7	311	41.1	364	49.6	377	50.3
Breastfeeding								
Never	542	71.6	537	68.8	484	64.8	461	60.0
Ever	215	28.4	244	31.2	263	35.2	307	40.0
Postmenopausal hormone use								
Never	573	75.9	592	75.9	520	69.8	562	73.2
Ever	182	24.1	188	24.1	225	30.2	206	26.8
First degree family history								
No	579	79.6	668	87.9	584	80.0	648	86.3
Yes	148	20.4	92	12.1	146	20.0	103	13.7
Menopausal status								
Premenopausal	179	24.1	206	27.4	292	40.0	295	40.3
Postmenopausal	565	75.9	547	72.6	438	60.0	438	59.8
Age At first birth & parity								
Nulliparous	93	12.3	88	11.3	104	13.9	82	10.7
First birth before age 21	107	14.1	127	16.3	90	12.1	101	13.2
First birth between 21- <25	253	33.4	266	34.1	229	30.7	279	36.3
First Birth Between 25- < 29	174	23.0	173	22.2	170	22.8	185	24.1
First birth between 29- < 32	78	10.3	74	9.5	85	11.4	68	8.9
First birth after age 32	52	6.9	53	6.8	68	9.1	53	6.9
***Continuous variables***	***Mean***	***SD***	***Mean***	***SD***	***Mean***	***SD***	***Mean***	***SD***
Age at menarche	12.6	1.6	12.6	1.7	12.5	1.5	12.6	1.7
Body mass index	27.4	6.0	25.3	7.2	25.7	5.2	26.1	5.7

In the LIBCSP population, 747 cases (49.5%) and 768 controls (49.4%) reported using a fireplace or indoor stove at least 3 times per year in one of their Long Island residences (Table 2). The predominant material burned in stoves/fireplaces was wood (44.8% of cases and 45.1% controls). The second most common fuel source used was synthetic logs, with 246 cases (16.4%) and 202 controls (13.0%) burning synthetic logs, followed by coal use (5.8% of cases and 4.9% of controls) and gas use (1.5% of cases and 1.4% of controls).

**Table 2:** **Association between indoor stove/fireplace use and breast cancer risk** ^**a**^

Exposure Status	Controls	Cases	Age-adjusted OR (95% CI)	Multivariable-adjusted OR (95% CI) ^b^
	N (%)	N (%)		
**Ever use of any indoor stove/fireplace** ^**c**^				
Never	781 (50.4)	757 (50.3)	1.00 (ref)	1.00 (ref)
Ever	768 (49.4)	747 (49.5)	1.05 (0.91, 1.21)	1.05 (0.88, 1.25)
**Material burned** ^**d**^				
None	781 (50.4)	757 (50.3)	1.00 (ref)	1.00 (ref)
Wood	699 (45.1)	674 (44.8)	0.93 (0.79, 1.08)	0.93 (0.77, 1.12)
Synthetic Logs	202 (13.0)	246 (16.4)	1.45 (1.17, 1.80)	1.42 (1.11, 1.84)
Coal	76 (4.9)	87 (5.8)	1.27 (0.92, 1.76)	1.28 (0.88, 1.86)
Gas	22 (1.4)	22 (1.5)	1.08 (0.59, 1.96)	0.86 (0.42, 1.73)

^a^Among Long Island, NY women, LIBCSP 1996-1997.

^b^Multivariate OR adjusted for age, age at menarche, history of breastfeeding, hormone therapy use, family history of breast cancer, parity, age at first birth, BMI at reference, education, smoking history, alcohol intake, physical activity, race, religion, marital status.

^c^Use of any indoor stove or fireplace regardless of the material used.

^d^Materials burned categories are not mutually exclusive. Wood burning excludes synthetic logs.

As shown in Table 2, breast cancer risk was increased by 42% in association with ever burning synthetic logs in the fireplace or indoor stove (OR = 1.42, 95% CI 1.11, 1.84). However, there was no increase in risk for burning wood (OR = 0.93, 95% CI 0.77, 1.12), or when we combined all types of residential indoor fireplace/stove exposure (OR = 1.05, 95% CI 0.88, 1.25). A non-significant association with breast cancer was observed for burning coal (OR = 1.28, 95% CI 0.88, 1.86). The variables representing women who reported burning wood and those burning synthetic logs were not strongly correlated and correlations were similar among cases (k = 0.28) and controls (k = 0.22).

As shown in Table 3, the increased breast cancer risk associated with burning synthetic logs indoors was apparent among women reporting 7+ years of use (p for linear trend <0.05). For burning synthetic logs, there was no association with breast cancer for ≤6.9 years of exposure (OR = 1.12, 95% CI 0.68, 1.82), but risk was increased for exposure of 7.0-16.7 years (OR = 1.73, 95% CI 1.11, 2.70) and for >24.8 years (OR = 1.50, 95% CI 0.98, 2.31). Among women who reported burning wood in their indoor stoves/fireplaces, there was a modest suggested increase in breast cancer risk for those between 21.6-31.0 years of exposure (OR = 1.20, 95% CI 0.92-1.55); however, this association was not evident for exposures occurring for more than 31 years (OR = 0.88, 95% CI 0.66, 1.17). When we considered any residential indoor stove/fireplace use, regardless of the material burned, no linear trend association with duration of use was noted. Study participant characteristics have been previously found to differ by whether or not the respondents reported living in the same home for 15 years or more [22]. However, a sensitivity analysis restricted to long-term Long Island residents (≥15 years) found a similar trend among any stove/fireplace and wood users and an increasing trend for women burning synthetic logs for 19 years or longer (see Additional file 1).

**Table 3:** **Association between duration (years) of indoor stove/fireplace use and breast cancer risk** ^**a**^

Exposure	Years of exposure	Controls	Cases	Age-adjusted OR	Multivariable-adjusted OR ^b^
		(N)	(N)	(95% CI)	(95% CI)
**Any indoor stove/fireplace use**	No stove/fireplace use	781	757	1.00 (reference)	1.00 (reference)
	≤11.6 years	190	183	1.13 (0.89, 1.43)	1.15 (0.87, 1.51)
	11.7-21.6 years	190	172	1.02 (0.81, 1.30)	1.00 (0.76, 1.32)
	21.7-30.7 years	190	208	1.18 (0.94, 1.47)	1.13 (0.87, 1.46)
	>30.7 years	190	181	0.93 (0.74, 1.17)	0.95 (0.72, 1.25)
**Wood burning** ^**c**^	No stove/fireplace use	781	757	1.00 (reference)	1.00 (reference)
	≤11.0 years	173	159	1.08 (0.84, 1.38)	1.07 (0.80, 1.42)
	11.1-21.4 years	173	156	1.03 (0.80, 1.31)	1.00 (0.75, 1.33)
	21.5-30.9 years	173	199	1.23 (0.97, 1.55)	1.20 (0.92, 1.55)
	>30.0 years	173	157	0.88 (0.69, 1.13)	0.88 (0.66, 1.17)
**Synthetic log burning**	No stove/fireplace use	781	757	1.00 (reference)	1.00 (reference)
	≤6.9 years	50	41	0.98 (0.63, 1.52)	1.12 (0.68, 1.82)
	7.0-16.7 years	50	69	1.69 (1.14, 2.50)	1.73 (1.11, 2.70)
	16.7-24.8 years	50	58	1.31 (0.88, 1.95)	1.29 (0.82, 2.03)
	>24.8 years	49	76	1.53 (1.05, 2.24)	1.50 (0.98, 2.31)

^a^Among Long Island, NY women, LIBCSP 1996-1997.

^b^Multivariate OR adjusted for age, age at menarche, history of breastfeeding, hormone therapy use,

family history of breast cancer, parity, age at first birth, BMI at reference, education, smoking history,

alcohol intake, physical activity, race, religion, marital status.

^c^Wood burning excludes synthetic logs.

As shown in Table 4, the increase in breast cancer risk associated with burning synthetic logs varied by the timing of the exposure. For example, stratifying by whether exposures occurred before or after 20 years of age, suggested that for synthetic logs, the increased risk is limited to the exposures occurring after age 20 years (OR = 1.65, 95% CI 1.02, 2.67), rather than exposure occurring prior to age 20 years (OR = 1.09, 95% CI 0.46, 2.59), which was imprecise due to large confidence intervals. In contrast, there was a suggested increase in breast cancer risk for exposure prior to 20 years of age for any stove/fireplace (OR = 1.28, 95% CI 0.89, 1.83) and for wood use (OR = 1.32, 0.91, 1.92), but no association for exposures occurring after age 20.

**Table 4:** **Association between age period of exposure** ^**a**^ **of stove/fireplace use and breast cancer risk** ^**b**^

Exposure timing	Controls	Cases	Age-adjusted OR (95% CI)	Multivariable-adjusted OR (95% CI) ^c^
	N (%)	N (%)		
**Any indoor stove/fireplace**				
Never	228	190	1.00 (reference)	1.00 (reference)
<20 years of age	196	173	1.09 (0.82, 1.45)	1.28 (0.89, 1.83)
≥20 years of age	171	146	1.05 (0.78, 1.40)	1.07 (0.75, 1.52)
**Wood burning** ^**d**^				
Never	228	190	1.00 (reference)	1.00 (reference)
<20 years of age	175	156	1.10 (0.83, 1.48)	1.32 (0.91, 1.92)
≥20 years of age	167	141	1.03 (0.76, 1.38)	1.05 (0.73, 1.50)
**Synthetic log burning**				
Never	228	190	1.00 (reference)	1.00 (reference)
<20 years of age	28	23	1.14 (0.63, 2.08)	1.09 (0.46, 2.59)
≥20 years of age	65	76	1.47 (1.00, 2.17)	1.65 (1.02, 2.67)

^a^Exposure timing = age (in years) when exposed to indoor air pollution (time periods are not mutually exclusive, see text).

^b^Among Long Island, NY women, 1996-1997.

^c^Multivariate OR adjusted for age, age at menarche, history of breastfeeding, hormone therapy use, family history of breast cancer, parity, age at first birth, BMI at reference, education, smoking history, alcohol intake, physical activity, race, religion, marital status.

^d^Wood burning excludes synthetic logs.

We did not observe any statistically evident heterogeneity by p53 mutation status (see Additional file 1). Associations between specific p53 mutation types are also available in the supplemental material (see Additional file 1); due to small sample sizes, estimates shown are adjusted for only the frequency-matching factor, age. While the associations are imprecise, these results may warrant further investigation. When we considered breast cancer tumor subtype as defined by ER/PR status, we found little heterogeneity in the ORs for the association with indoor stove/fireplace use (data not shown).

As shown in Table 5, the association between synthetic log burning and breast cancer risk increased with number of GST variants; women with 2+ GST variants and who burned synthetic logs had a significant increased risk (OR = 1.71, 95% CI 1.09, 2.68). However, we observed no statistical effect modification by number of GST variants or with each individual GST variant (see Table 5; see Additional file 1). Further, there was no effect modification observed by menopausal status or active/passive smoking (data not shown).

**Table 5:** **Interaction between the number of variant** ***GST*** ^**a**^ **genotypes and indoor stove/fireplace use and breast cancer** ^**b**^

Number of GST ^a^ variants	Indoor stove/ fireplace use	Cases (n)	Controls (n)	Age-adjusted OR (95% CI)	Multivariable-adjusted OR (95% CI) ^c^
	**Ever any**				
<2 variants	No stove/fireplace	452	483	1.00 (reference)	1.00 (reference)
	Ever any stove/fireplace	424	419	1.06 (0.96, 1.16)	1.04 (0.93, 1.16)
≥2 variants	No stove/fireplace	305	298	1.12 (0.93, 1.35)	1.08 (0.87, 1.35)
	Ever any stove/fireplace	323	349	1.19 (0.89, 1.58)	1.13 (0.81, 1.57)
	**Wood burning** ^**d**^				
<2 variants	No stove/fireplace	452	483	1.00 (reference)	1.00 (reference)
	Ever wood burning	378	377	1.05 (0.96, 1.16)	1.02 (0.91, 1.15)
≥2 variants	No stove/fireplace	305	298	1.11 (0.92, 1.35)	1.05 (0.83, 1.32)
	Ever wood burning	296	322	1.17 (0.88, 1.57)	1.07 (0.76, 1.51)
	**Synthetic log burning**				
<2 variants	No stove/fireplace	452	483	1.00 (reference)	1.00 (reference)
	Ever synthetic log	146	112	1.22 (1.07, 1.38)	1.19 (1.03, 1.39)
≥2 variants	No stove/fireplace	305	298	1.48 (1.15, 1.91)	1.43 (1.06, 1.93)
	Ever synthetic log	100	90	1.81 (1.24, 2.63)	1.71 (1.09, 2.68)

^a^Number of GST variants = total number of variant genotypes from any of the GST SNPs considered in this study, including*: GSTA1*, *GSTT1*, *GSTM1*, *GSTP1* (see text). Common alleles: GSTT1 present, GSTM1 present, GSTA1*A, GSTP1 (AA). Variant alleles: GSTT1 null, GSTM1 null, GSTA1*B, GSTP1 (AG or GG).

^b^Among Long Island, NY women, LIBCSP, 1996-1997.

^c^Multivariate OR adjusted for age, age at menarche, history of breastfeeding, hormone therapy use,

family history of breast cancer, parity, age at first birth, BMI at reference, education, smoking history,

alcohol intake, physical activity, race, religion, marital status.

^d^Wood burning excludes synthetic logs.

## Discussion

In this population-based study, we found that women who burned synthetic logs at a Long Island residence were 45% more likely to have breast cancer compared to women who did not. This association appeared to be more pronounced for women who burned synthetic logs in their home for >7 years, for those exposed during their adult years or for those with multiple GST variant alleles. However, there was no association observed with wood burning in the home, or when all types of stove/fireplace burning material were considered together. Given that indoor exposure to PAHs occurs with burning either wood or synthetic logs but our findings were limited to synthetic logs, our results should be interpreted with care, as discussed below, and require replication.

To the best of our knowledge, this is the first study to examine the association between breast cancer risk and an important source of ambient PAH exposure, the use of indoor stoves/fireplaces, an indicator of indoor air pollution [34]. Our results have public health significance, given that a recent scientific review concluded that “estimating the effects of household solid-fuel combustion on cancers other than lung” and whether “genetic susceptibility modifies” the association were high priority research topics [11].

Use of solid fuels for indoor heating and cooking is most common in Africa and Southeast Asia, with a prevalence of use at approximately 60% and thus, most research on health impacts have been conducted in countries in these geographic areas [11, 35]. However, solid fuel remains as the primary heating source for approximately 6.5 million U.S. citizens, predominately those of low socioeconomic status [36], but may be used recreationally or as a supplemental heating source among non-low income homes [37]. Most stoves in the U.S. tend to only be used seasonally and have a flue to remove smoke from the home [36]. While we do not have measurements of indoor air concentrations from the LIBCSP, one study conducted in a Swedish residential area found wood burning homes had a median benzo[a]pyrene (BaP) level of 0.52 ng/m^3^[34]; orders of magnitude lower than the mean BaP in wood burning homes in India, 0.70 ug/m^3^ (equivalent to 700 ng/m^3^) [38]. This large discrepancy is likely due to the stove/fireplace type, limited ventilation and increased year-round duration of use. Overall, these differences suggest that exposure levels to PAHs from stoves/fireplaces would be likely lower in countries like the U.S. The association observed with breast cancer risk in this study with lower exposure levels to stove/fireplace use, suggest that if confirmed, this association may be even stronger in low or middle income countries where exposures are much higher. However, to the best of our knowledge no other study has investigated this research question either in the U.S. or elsewhere.

There has been limited testing on the emission profiles of synthetic logs, consisting of wax and sawdust, compared to natural logs. The increase in breast cancer risk we observed with synthetic log burning may be biologically plausible. A report by Gullet et al. [39] measured emission factors of various pollutants (including polychlorinated dibenzodioxins and dibenzofurans, polychlorinated biphenyls, hexacholorbenzene, particulate matter and PAHs) from fireplace and woodstove combustion emissions. Synthetic log burning produced elevated levels of almost all measured PAHs compared to the combustion of wood, but not higher levels of the other pollutants measured [39]. Specific PAHs that were markedly high in concentration in the synthetic log emissions included chrysene/triphenylene, benzo(e)pyrene and retene [39]. Chrysene in particular is a documented tumorigenic PAH [2]. Two other emission analysis also reported higher PAH levels and a wider range of specific PAH compounds present in synthetic log emissions compared to wood emissions [40, 41]. These results suggest that PAHs may be one of the more relevant emission factors from synthetic log burning for breast cancer risk. However, other reports have found similar PAH levels across synthetic logs and natural wood logs or possibly lower PAH levels for synthetic logs [42, 43]. These inconsistencies could be due to differing synthetic log components or testing conditions, and thus variable emission characteristics [44].

Retrospective stove/fireplace use was collected in the mid-1990’s which would have reflected exposures before the Environmental Protection Agency began endorsing certified indoor stoves/fireplaces with lower emissions [45]. This, combined with the recent changes in composition of synthetic logs away from using petroleum wax [44], may result in this study population having higher exposure levels than those that exist today.

A Washington state department of health study estimated that 40% of people used some type of wood burning device (lower than the ~50% prevalence observed here) [46]. Among the Washington users of wood burning devices, approximately 50% used woodstoves, 50% used fireplaces and 17% used both devices [46]. Synthetic logs are manufactured to be used in open fireplaces, which tend to emit more PAHs than wood stoves per hour of operation [47]. In fact, an open fireplace burning natural wood can result in PAH concentrations comparable to those of ambient urban air [48]. Open fireplace use, but not indoor stove use, has also been associated with increased DNA adduct levels in both mothers and their newborns [14]. Thus, it is possible that the effect seen here is a surrogate measure for open fireplace use (regardless of material) as synthetic logs are not recommended to be used in indoor stoves. Collapsing indoor stove and fireplace use into one question is a limitation of this study questionnaire, and may have resulted in an attenuation of the result observed for wood use in the home. This interpretation would rely on natural wood being predominately burned in indoor stoves, which are generally closed and designed to be airtight, and thus, may release fewer emissions [47]. This hypothesis should be further investigated in future research.

PAHs may have both mutagenic and epigenetic possible mechanisms that are relevant to breast carcinogenesis [2]. PAHs can be rapidly absorbed into the human body after inhalation of indoor air pollution from indoor stoves/fireplaces, and are soon released into the general circulation [5]. PAHs are lipophilic and can accumulate in adipose tissue of the breast [8]. Phase 1 enzymes break down PAHs to reactive metabolites which can then be detoxified by Phase II metabolic pathways (e.g., the GSTs) [3]. When the exposure levels are high, or detoxification processes are not adequate, PAH metabolites can bind to DNA to form adducts [3]. If DNA repair processes are unable to rectify this DNA damage, it can ultimately lead to somatic mutations in breast cancer-related genes, an initiation step of carcinogenesis [2].

We did find a suggestive evidence that any indoor stove/fireplace use and wood burning prior to age 20 years, which encompasses age before first birth for approximately 90% of the LIBCSP study population, may be more relevant for future breast cancer risk than exposure that occurs after age 20 years. This is consistent with evidence from another PAH source, active smoking, which may be particularly important to breast cancer risk if the exposure is prior to the first pregnancy [49]. On the contrary, we observed that breast cancer risk was higher for exposure to synthetic log use after age 20 years. Synthetic logs were commercialized in 1931 with the petroleum-based synthetic logs being introduced in 1963 [50]. Thus, we cannot rule out the possibility that the older age of our population precluded many from being exposed to synthetic logs at an early age resulting in small sample sizes and unstable estimates. We are unable to take into account any stove/fireplace exposures from participant’s former residences that were not on Long Island due to the design of the original study questionnaire. While many in the study population have been long-term residents of Long Island [58% had lived in their current Long Island residence for 15 or more years [22], this makes it particularly challenging to estimate the effect of early life exposures from indoor stove/fireplace use.

Reporting the use of indoor stoves/fireplaces is a surrogate measure of indoor PAH exposure, but other factors (such as exterior air pollution from vehicular traffic in urban areas) may contribute to indoor air pollution levels [2]. Therefore, we cannot rule out the possibility of other PAH sources affecting indoor air pollution levels. Stove/fireplaces may also release pollutants in addition to PAHs that may be relevant to breast cancer risk [11]. For example, synthetic logs may emit polychlorinated biphenyls, particulate matter, polychlorinated dibenzodioxins and dibenzofurans, hexacholorbenzene, carbon monoxide, nitrogen oxides, volatile organic compounds and formaldehyde [39, 43, 44]. However, none of these chemicals have been found to be consistently associated with breast cancer risk as is the case for PAHs across different exposure sources [51]. In addition, although we did assess duration of exposure, we did not assess frequency of exposure to indoor stove/fireplace burning, which makes identifying and quantifying the association with those who are most highly exposed very difficult. One report found that those who burned synthetic logs did so relatively frequently (at least several times per month) [39]. Having information on frequency of use would better inform the results reported here.

We did not find an association between stove/fireplace use and p53-mutation-positive tumors, which is consistent with previous results from the LIBCSP investigating other ambient PAH sources [52], but contrasts with other study populations in which cigarette smoking has been found to be associated with tumor p53 mutation status [53, 54]. This discrepancy could, among other potential factors, be due to intrinsic differences in the PAH sources being measured.

We observed no evidence for an interaction between individual variant GST alleles with indoor stove/fireplace use, despite the biologically plausibility [19]. However, there was some suggestion that multiple GST variant alleles interacted with synthetic log burning to increase breast cancer risk. Our results are consistent with a previous report investigating GST interactions with other multiple PAH sources in Long Island [20]. Both specific GSTs and number of GST variants have been previously associated with breast cancer in the LIBCSP and in other study populations, although results have been inconsistent [24, 25, 55, 56].

Differential recall bias is always a potential concern for case-control studies. However, our most prominent finding is among those that burned synthetic logs in their homes. It is unlikely at the time of data collection that cases would have suspected certain wood-based fuel materials would be more carcinogenic than others. In addition, this study population had many long-term users of synthetic logs which suggest these results are not just an artifact of differential recall. Despite this, it is still possible that this association may have been impacted by recall bias. There is also the possibility of the influence of selection bias, particularly among older control women who had a lower response rate. However, the mechanism by which stove/fireplace use would affect response rates among only elderly control women is unknown. Participations rates may vary by socioeconomic status. High socioeconomic status is a risk factor for breast cancer [1]; however, indoor stove/fireplace use is more common in low-income populations [36]. Thus, it is unlikely that stove/fireplace use is a proxy for socioeconomic status. There was also little existing literature on predictors of stove/fireplace use. Therefore, residual confounding may be present, either by the lack of inclusion of a confounder or imprecise measurement of a confounder included in the model. However, we included many known breast cancer risk factors in our adjustment sets in order to mitigate this concern. Another study concern is the low prevalence of some specific p53 mutations in our population such that, despite our fairly large sample size, we were unable to more precisely estimate the association of burning either coal or gas, or the associations with p53-defined breast cancer subtype. While imprecise and difficult to interpret, we believe these results could be useful for future studies when considering environmental exposures such as PAHs and breast cancer risk. The novel result of an increased breast cancer risk associated with synthetic log use, while biologically plausible, does require replication in other study population.

## Conclusions

Our findings suggest that residential indoor air pollution from burning synthetic logs, but not wood, may be associated with an increase in breast cancer risk. The high incidence of breast cancer in the U.S. and the relatively common prevalence of indoor stoves/fireplaces suggest that this research, if confirmed, may have considerable public health importance. Our results provides new information on an important ambient PAH exposure source, which has been understudied in populations with low exposure levels such as those found in the U.S., and may guide future research on the potential carcinogenicity of PAH exposure on the breast.

## Electronic supplementary material

Additional file 1: **Indoor Air Pollution Exposure from Use of Indoor Stoves and Fireplaces in Association with Breast Cancer: A Case-Control Study.** (DOCX 43 KB)

## Abbreviations

(95%CI): 95% confidence interval
(ERPR): Estrogen receptor progesterone receptor
(GST): Glutathione S-transferase
(IARC): International Agency for Research on Cancer
(ICR): Interaction contrast ratio
(LIBCSP): Long Island Breast Cancer Study Project
(OR): Odds ratio
(PAH): Polycyclic aromatic hydrocarbons
(U.S.): United States.
