# Supplementary material for: Indoor air pollution exposure from use of indoor stoves and fireplaces in association with breast cancer: a case-control study
Source: Environ Health. 2014 Dec 12;13:108. doi: 10.1186/1476-069X-13-108 (PMC4320487; doi:10.1186/1476-069X-13-108)
Supplement: Supplementary file 1 — Additional file 1: Indoor Air Pollution Exposure from Use of Indoor Stoves and Fireplaces in Association with Breast Cancer: A Case-Control Study. (DOCX 43 KB) [file 12940_2014_826_MOESM1_ESM.docx]

**Supplemental Material for:**

**Alexandra J. White, et al., Indoor Air Pollution Exposure from Use of Indoor Stoves and Fireplaces in Association with Breast Cancer: A Case-Control Study**

**Supplemental Table I.** Multivariable and age-adjusted odds ratios (OR) and 95% confidence intervals (CI) for the association between duration (years) of indoor stove/fireplace use and breast cancer risk among long-term Long Island residents (≥15 years) among Long Island, NY women, LIBCSP, 1996-1997

| **Exposure** | **Years of exposure** | **Controls** | **Cases** | **Age-adjusted OR** | **Multivariable-adjusted OR^c^** |
| --- | --- | --- | --- | --- | --- |
|  |  | **(N)** | **(N)** | **(95% CI)** | **(95% CI)** |
| **Any indoor stove/fireplace use** | No stove/fireplace use | 632 | 616 | 1.00 (reference) | 1.00 (reference) |
|  | ≤14.6 years | 168 | 140 | 0.99 (0.76, 1.28) | 0.99 (0.73, 1.33) |
|  | 14.7-23.2 years | 169 | 163 | 1.08 (0.84, 1.29) | 1.13 (0.85, 1.51) |
|  | 23.3-31.6 years | 162 | 182 | 1.22 (0.95, 1.55) | 1.15 (0.87, 1.52) |
|  | >31.6 years | 174 | 165 | 0.93 (0.73, 1.18) | 0.92 (0.69, 1.23) |
| **Wood burning^b^** | No stove/fireplace use | 632 | 616 | 1.00 (reference) | 1.00 (reference) |
|  | <14.6 years | 154 | 124 | 0.95 (0.73, 1.25) | 0.95 (0.70, 1.29) |
|  | 14.6-23.2 years | 148 | 139 | 1.12 (0.89, 1.40) | 1.13 (0.87, 1.48) |
|  | 23.2-31.6 years | 137 | 157 | 1.24 (0.95, 1.61) | 1.20 (0.90, 1.62) |
|  | >31.6 years | 160 | 146 | 0.89 (0.69, 1.15) | 0.88 (0.65, 1.19) |
| **Synthetic log burning** | No stove/fireplace use | 632 | 616 | 1.00 (reference) | 1.00 (reference) |
|  | ≤ 9.8 years | 63 | 64 | 0.93 (0.57, 1.60) | 1.01 (0.55, 1.86) |
|  | 9.8-19.0 years | 48 | 59 | 0.97 (0.82, 1.15) | 0.99 (0.81, 1.20) |
|  | 19.1-25.6 | 44 | 49 | 1.28 (0.86, 1.91) | 1.23 (0.77, 1.96) |
|  | > 25.6 years | 44 | 72 | 1.57 (1.07, 2.31) | 1.45 (0.93, 2.25) |

^a^Multivariate OR adjusted for age, age at menarche, history of breastfeeding, hormone therapy use, family history of breast cancer, parity, age at first birth, BMI at reference, education, smoking history, alcohol intake, physical activity, race, religion, marital status

^b^Wood burning excludes synthetic logs.

**Supplemental Table II.** Multivariable and age-adjusted odds ratios (OR) and 95% confidence intervals (CI) for the association between indoor stove/fireplace use and breast cancer subtype as defined by p53 mutation status among Long Island, NY women, LIBCSP, 1996-1997.^a^

|  |  | ***p53+ Cases*** | | | ***p53- Cases*** | | | **Ratio of the ORs (95%CI)**  ***p53+* vs. *p53-*** | **Ratio of the ORs (95%CI)^b^**  ***p53+* vs. *p53-*** |
| --- | --- | --- | --- | --- | --- | --- | --- | --- | --- |
| **Indoor Air Pollution** | **Co** | **Ca** | **Age-Adjusted**  **OR (95%CI)** | **Multivariable-Adjusted^c^**  **OR (95%CI)** | **Ca** | **Age-Adjusted**  **OR (95%CI)** | **Multivariable-Adjusted^c^**  **OR (95%CI)** |  |  |
| Any indoor stove/fireplace |  |  |  |  |  |  |  |  |  |
| No stove/fireplace | 781 | 74 | 1.00 (reference) | 1.00 (reference) | 367 | 1.00 (reference) | 1.00 (reference) | 1.00 (reference) | 1.00 (reference) |
| Ever stove/fireplace | 768 | 54 | 0.78 (0.53, 1.13) | 0.76 (0.47, 1.21) | 361 | 1.06 (0.89, 1.28) | 1.04 (0.84, 1.31) | 0.75 (0.50, 1.10) | 0.87 (0.52, 1.46) |
| Wood burning^c^ |  |  |  |  |  |  |  |  |  |
| No stove/fireplace | 781 | 74 | 1.00 (reference) | 1.00 (reference) | 367 | 1.00 (reference) | 1.00 (reference) | 1.00 (reference) | 1.00 (reference) |
| Ever wood burning | 699 | 48 | 0.75 (0.50, 1.10) | 0.77 (0.48, 1.24) | 323 | 1.05 (0.87, 1.26) | 0.87 (0.78, 1.21) | 0.73 (0.49, 1.09) | 0.88 (0.52, 1.47) |
| Synthetic log burning |  |  |  |  |  |  |  |  |  |
| No stove/fireplace | 781 | 74 | 1.00 (reference) | 1.00 (reference) | 367 | 1.00 (reference) | 1.00 (reference) | 1.00 (reference) | 1.00 (reference) |
| Ever synthetic logs | 202 | 17 | 1.05 (0.59, 1.87) | 1.07 (0.53, 2.15) | 121 | 1.43 (1.10, 1.87) | 1.56 (1.17, 2.10) | 0.73 (0.41, 1.33) | 0.84 (0.41, 1.72) |

^a^ Ca= cases Co=controls; *p53+* = case with any *p53* mutation; *p53-* = case without any *p53* mutation (see text).

**^b^** Multivariate OR adjusted for age, age at menarche, history of breastfeeding, hormone therapy use, family history of breast cancer, parity, age at first birth, BMI at reference, education, smoking history, alcohol intake, physical activity, race, religion, marital status

^c^ Wood burning excludes synthetic logs.

**Supplemental Table III.** Age-adjusted odds ratios (ORs) and 95% confidence intervals (CI) for the association between indoor stove/fireplace use and breast cancer defined by p53 mutation type among Long Island, NY women, LIBCSP, 1996-1997.^a^

| **p53 Mutation Type** | **Indoor Air Pollution Exposure** | **Cases with no p53 mutations (n)** | **Cases with specific p53 mutation type**  **(n)** | **Age-adjusted OR (95% CI)** |
| --- | --- | --- | --- | --- |
| **G:C 🡪 A:T at CpG transition** |  |  |  |  |
|  | **Ever use of any indoor stove/fireplace** |  |  |  |
|  | No stove/fireplace use | 781 | 18 | 1.00 (reference) |
|  | Ever any stove/fireplace use | 768 | 16 | 0.90 (0.45, 1.80) |
|  | **Burned wood** |  |  |  |
|  | No stove/fireplace use | 781 | 18 | 1.00 (reference) |
|  | Ever wood burning | 699 | 14 | 0.87 (0.43, 1.78) |
|  | **Burned synthetic logs** |  |  |  |
|  | No stove/fireplace use | 781 | 18 | 1.00 (reference) |
|  | Ever synthetic logs | 202 | 7 | 1.67 (0.66, 4.16) |
| **G:C 🡪 A:T at non-CpG transition** |  |  |  |  |
|  | **Ever use of any indoor stove/fireplace** |  |  |  |
|  | No stove/fireplace use | 781 | 25 | 1.00 (reference) |
|  | Ever any stove/fireplace use | 768 | 17 | 0.72 (0.38, 1.37) |
|  | **Burned wood** |  |  |  |
|  | No stove/fireplace use | 781 | 25 | 1.00 (reference) |
|  | Ever wood burning | 699 | 13 | 0.61 (0.31, 1.23) |
|  | **Burned synthetic logs** |  |  |  |
|  | No stove/fireplace use | 781 | 25 | 1.00 (reference) |
|  | Ever synthetic logs | 202 | 5 | 0.91 (0.33, 2.51) |
| **Insertions/**  **Deletions** |  |  |  |  |
|  | **Ever use of any indoor stove/fireplace** |  |  |  |
|  | No stove/fireplace use | 781 | 17 | 1.00 (reference) |
|  | Ever any stove/fireplace use | 768 | 9 | 0.56 (0.25, 1.30) |
|  | **Burned wood** |  |  |  |
|  | No stove/fireplace use | 781 | 17 | 1.00 (reference) |
|  | Ever wood burning | 699 | 8 | 0.54 (0.23, 1.28) |
|  | **Burned synthetic logs** |  |  |  |
|  | No stove/fireplace use | 781 | 17 | 1.00 (reference) |
|  | Ever synthetic logs | 202 | <5 | N/A^b^ |

^a^Using a case-case analysis approach (comparing target cases (women with a specific p53 mutation in their breast tumor) vs. comparison cases (women without any p53 mutation in their breast tumor)

^b^N/A= not ascertained, given the cell size <5

**Supplemental Table IV**. Odds ratios (ORs) and 95% confidence intervals (CI) for the association between indoor stove/fireplace use and breast cancer defined by p53 mutation effect among Long Island, NY women, LIBCSP, 1996-1997.^a^

| **Mutation Effect** | **Exposure** | **Cases with no p53 mutation (n)** | **Cases with specific p53 mutation type**  **(n)** | **Age-adjusted OR (95% CI)** |
| --- | --- | --- | --- | --- |
| **Missense** |  |  |  |  |
|  | **Ever use of any indoor stove/fireplace** |  |  |  |
|  | No stove/fireplace use | 781 | 37 | 1.00 (reference) |
|  | Ever any stove/fireplace use | 768 | 33 | 0.93 (0.57, 1.52) |
|  | **Burned wood** |  |  |  |
|  | No stove/fireplace use | 781 | 37 | 1.00 (reference) |
|  | Ever wood burning | 699 | 29 | 0.90 (0.54, 1.49) |
|  | **Burned synthetic logs** |  |  |  |
|  | No stove/fireplace use | 781 | 37 | 1.00 (reference) |
|  | Ever synthetic logs | 202 | 10 | 1.23 (0.58, 2.60) |
| **Nonsense** |  |  |  |  |
|  | **Ever use of any indoor stove/fireplace** |  |  |  |
|  | No stove/fireplace use | 781 | 10 | 1.00 (reference) |
|  | Ever any stove/fireplace use | 768 | 6 | 0.60 (0.21, 1.71) |
|  | **Burned wood** |  |  |  |
|  | No stove/fireplace use | 781 | 10 | 1.00 (reference) |
|  | Ever wood burning | 699 | 5 | 0.55 (0.18, 1.67) |
|  | **Burned synthetic logs** |  |  |  |
|  | No stove/fireplace use | 781 | 10 | 1.00 (reference) |
|  | Ever synthetic logs | 202 | <5 | N/A^c^ |
| **Silent** |  |  |  |  |
|  | **Ever use of any indoor stove/fireplace** |  |  |  |
|  | No stove/fireplace use | 781 | 16 | 1.00 (reference) |
|  | Ever any stove/fireplace use | 768 | 8 | 0.58 (0.24, 1.39) |
|  | **Burned wood** |  |  |  |
|  | No stove/fireplace use | 781 | 16 | 1.00 (reference) |
|  | Ever wood burning | 699 | 6 | 0.48 (0.18, 1.25) |
|  | **Burned synthetic logs** |  |  |  |
|  | No stove/fireplace use | 781 | 16 | 1.00 (reference) |
|  | Ever synthetic logs | 202 | <5 | N/A^c^ |
| **Frameshift mutation** |  |  |  |  |
|  | **Ever use of any indoor stove/fireplace** |  |  |  |
|  | No stove/fireplace use | 781 | 17 | 1.00 (reference) |
|  | Ever any stove/fireplace use | 768 | 9 | 0.56 (0.25, 1.29) |
|  | **Burned wood** |  |  |  |
|  | No stove/fireplace use | 781 | 17 | 1.00 (reference) |
|  | Ever wood burning | 699 | 8 | 0.54 (0.23, 1.27) |
|  | **Burned synthetic logs** |  |  |  |
|  | No stove/fireplace use | 781 | 17 | 1.00 (reference) |
|  | Ever synthetic logs | 202 | <5 | N/A^b^ |

^a^Using a case-case analysis approach (comparing target cases (women with a specific p53 mutation in their breast tumor) vs. comparison cases (women without any p53 mutation in their breast tumor)

^b^N/A= not ascertained, given the cell size <5**Supplemental Table V.** Odds ratios (ORs) and 95% confidence intervals (CI) for the association between any indoor stove/fireplace use and breast cancer by GST polymorphism among Long Island, NY women, LIBCSP 1996-1997.

| **GST Genotype** | **Any Indoor Stove/**  **Fireplace Use** | **Cases**  **(n)** | **Controls (n)** | **Age-adjusted**  **OR (95% CI)** | **Multivariable-adjusted OR (95%CI)^a^** |
| --- | --- | --- | --- | --- | --- |
| GSTT1 |  |  |  |  |  |
| Present | No stove/fireplace use | 379 | 375 | 1.00 (reference) | 1.00 (reference) |
|  | Ever any stove/fireplace use | 395 | 417 | 1.01 (0.91, 1.12) | 1.01 (0.90, 1.14) |
| Null | No stove/fireplace use | 106 | 116 | 0.97 (0.83, 1.13) | 0.91 (0.76, 1.10) |
|  | Ever any stove/fireplace use | 101 | 103 | 1.02 (0.87, 1.19) | 0.94 (0.78, 1.13) |
| GSTM1 |  |  |  |  |  |
| Present | No stove/fireplace use | 262 | 279 | 1.00 (reference) | 1.00 (reference) |
|  | Ever any stove/fireplace use | 241 | 264 | 1.01 (0.88, 1.14) | 1.05 (0.90, 1.22) |
| Null | No stove/fireplace use | 222 | 202 | 1.07 (0.94, 1.21) | 1.14 (0.99, 1.32) |
|  | Ever any stove/fireplace use | 248 | 251 | 1.07 (0.95, 1.21) | 1.09 (0.93, 1.27) |
| GSTP1 |  |  |  |  |  |
| AA (common) | No stove/fireplace use | 266 | 256 | 1.00 (reference) | 1.00 (reference) |
|  | Ever any stove/fireplace use | 249 | 261 | 1.00 (0.89, 1.13) | 0.98 (0.85, 1.13) |
| AG or GG | No stove/fireplace use | 247 | 266 | 0.95 (0.84, 1.08) | 0.90 (0.78, 1.040 |
|  | Ever any stove/fireplace use | 262 | 282 | 0.98 (0.87, 1.11) | 0.97 (0.83, 1.11) |
| GSTA1 |  |  |  |  |  |
| A*/A* | No stove/fireplace use | 178 | 205 | 1.00 (reference) | 1.00 (reference) |
|  | Ever any stove/fireplace use | 165 | 179 | 1.06 (0.91, 1.23) | 1.05 (0.88, 1.26) |
| A*/B* or B*/B* | No stove/fireplace use | 328 | 341 | 1.08 (0.95, 1.23) | 1.08 (0.93, 1.26) |
|  | Ever any stove/fireplace use | 373 | 354 | 1.08 (0.95, 1.24) | 1.09 (0.94, 1.27) |

^a^Multivariate OR adjusted for age, age at menarche, history of breastfeeding, hormone therapy use, family history of breast cancer, parity, age at first birth, BMI at reference, education, smoking history, alcohol intake, physical activity, race, religion, marital status

**Supplemental Table VI.** Odds ratios (ORs) and 95% confidence intervals (CI) for the association between wood log burning and breast cancer by GST polymorphism among Long Island, NY women, LIBCSP 1996-1997.

| **Genotype** | **Wood Log Burning** | **Cases (n)** | **Controls (n)** | **Age-adjusted**  **OR (95% CI)** | **Multivariable-adjusted OR (95%CI)^a^** |
| --- | --- | --- | --- | --- | --- |
| GSTT1 |  |  |  |  |  |
| Present | No stove/fireplace use | 379 | 375 | 1.00 (reference) | 1.00 (reference) |
|  | Ever wood burning | 360 | 374 | 1.02 (0.92, 1.13) | 1.02 (0.90, 1.15) |
| Null | No stove/fireplace use | 106 | 116 | 0.97 (0.83, 1.13) | 0.92 (0.77, 1.10) |
|  | Ever wood burning | 90 | 99 | 0.98 (0.83, 1.15) | 0.94 (0.78, 1.14) |
| GSTM1 |  |  |  |  |  |
| Present | No stove/fireplace use | 262 | 279 | 1.00 (reference) | 1.00 (reference) |
|  | Ever wood burning | 215 | 243 | 0.97 (0.84, 1.10) | 1.02 (0.88, 1.19) |
| Null | No stove/fireplace use | 222 | 202 | 1.07 (0.94, 1.21) | 1.14 (0.99, 1.32) |
|  | Ever wood burning | 229 | 226 | 1.08 (0.95, 1.22) | 1.12 (0.97, 1.30) |
| GSTP1 |  |  |  |  |  |
| AA (common) | No stove/fireplace use | 266 | 256 | 1.00 (reference) | 1.00 (reference) |
|  | Ever wood burning | 223 | 235 | 1.00 (0.88, 1.13) | 0.98 (0.85, 1.14) |
| AG or GG | No stove/fireplace use | 247 | 266 | 0.95 (0.84, 1.07) | 0.90 (0.79, 1.04) |
|  | Ever wood burning | 237 | 261 | 1.08 (0.95, 1.23) | 0.95 (0.82, 1.10) |
| GSTA1 |  |  |  |  |  |
| A*/A* | No stove/fireplace use | 178 | 205 | 1.00 (reference) | 1.00 (reference) |
|  | Ever wood burning | 144 | 161 | 1.04 (0.89, 1.22) | 1.04 (0.86, 1.25) |
| A*/B* or B*/B* | No stove/fireplace use | 328 | 341 | 1.08 (0.95, 1.23) | 1.08 (0.93, 1.26) |
|  | Ever wood burning | 343 | 323 | 1.08 (0.94, 1.23) | 1.08 (0.93, 1.27) |

^a^Multivariate OR adjusted for age, age at menarche, history of breastfeeding, hormone therapy use, family history of breast cancer, parity, age at first birth, BMI at reference, education, smoking history, alcohol intake, physical activity, race, religion, marital status

**Supplemental Table VII.** Odds ratios (ORs) and 95% confidence intervals (CI) for the association between synthetic log burning and breast cancer by GST polymorphism among Long Island, NY women, LIBCSP 1996-1997.

| **Genotype** | **Synthetic Log Burning** | **Cases (n)** | **Controls (n)** | **Age-adjusted OR (95% CI)** | **Multivariable-adjusted OR (95%CI)^a^** |
| --- | --- | --- | --- | --- | --- |
| GSTT1 |  |  |  |  |  |
| Present | No stove/fireplace use | 379 | 375 | 1.00 (reference) | 1.00 (reference) |
|  | Ever synthetic logs | 128 | 102 | 1.19 (1.04, 1.36) | 1.18 (1.00, 1.39) |
| Null | No stove/fireplace use | 106 | 116 | 0.97 (0.83, 1.13) | 0.93 (0.78, 1.12) |
|  | Ever synthetic logs | 34 | 28 | 1.19 (0.94, 1.51) | 1.07 (0.79, 1.45) |
| GSTM1 |  |  |  |  |  |
| Present | No stove/fireplace use | 262 | 279 | 1.00 (reference) | 1.00 (reference) |
|  | Ever synthetic logs | 82 | 56 | 1.32 (1.12, 1.54) | 1.37 (1.13, 1.67) |
| Null | No stove/fireplace use | 222 | 202 | 1.07 (0.94, 1.21) | 1.14 (0.98, 1.32) |
|  | Ever synthetic logs | 78 | 70 | 1.15 (0.97, 1.38) | 1.12 (0.90, 1.40) |
| GSTP1 |  |  |  |  |  |
| AA (common) | No stove/fireplace use | 266 | 256 | 1.00 (reference) | 1.00 (reference) |
|  | Ever synthetic logs | 87 | 65 | 1.21 (1.03, 1.42) | 1.20 (0.99, 1.46) |
| AG or GG | No stove/fireplace use | 247 | 266 | 0.95 (0.84, 1.07) | 0.91 (0.79, 1.05) |
|  | Ever synthetic logs | 85 | 72 | 1.14 (0.96, 1.35) | 1.08 (0.88, 1.32) |
| GSTA1 |  |  |  |  |  |
| A*/A* | No stove/fireplace use | 178 | 205 | 1.00 (reference) | 1.00 (reference) |
|  | Ever synthetic logs | 57 | 43 | 1.28 (1.05, 1.56) | 1.28 (1.01, 1.62) |
| A*/B* or B*/B* | No stove/fireplace use | 328 | 341 | 1.08 (0.95, 1.23) | 1.10 (0.95, 1.27) |
|  | Ever synthetic logs | 116 | 97 | 1.26 (1.07, 1.48) | 1.26 (1.03, 1.54) |

^a^Multivariate OR adjusted for age, age at menarche, history of breastfeeding, hormone therapy use, family history of breast cancer, parity, age at first birth, BMI at reference, education, smoking history, alcohol intake, physical activity, race, religion, marital status
